# A Morbidity Survey of South African Primary Care

**DOI:** 10.1371/journal.pone.0032358

**Published:** 2012-03-16

**Authors:** Bob Mash, Lara Fairall, Olubunmi Adejayan, Omozuanvbo Ikpefan, Jyoti Kumari, Shaheed Mathee, Ronit Okun, Willy Yogolelo

**Affiliations:** 1 Division of Family Medicine and Primary Care, Stellenbosch University, Cape Town, Western Cape, South Africa; 2 Knowledge Translation Unit, Lung Institute, University of Cape Town, Cape Town, Western Cape, South Africa; California Pacific Medical Center Research Institute, United States of America

## Abstract

**Background:**

Recent studies have described the burden of disease in South Africa. However these studies do not tell us which of these conditions commonly present to primary care providers, how these conditions may present and how providers make sense of them in terms of their diagnoses. Clinical nurse practitioners are the main primary care providers and need to be better prepared for this role. This study aimed to determine the range and prevalence of reasons for encounter and diagnoses found among ambulatory patients attending public sector primary care facilities in South Africa.

**Methodology/Principal Findings:**

The study was a multi-centre prospective cross-sectional survey of consultations in primary care in four provinces of South Africa: Western Cape, Limpopo, Northern Cape and North West. Consultations were coded prior to analysis by using the International Classification of Primary Care-Version 2 in terms of reasons for encounter (REF) and diagnoses. Altogether 18856 consultations were included in the survey and generated 31451 reasons for encounter (RFE) and 24561 diagnoses. Women accounted for 12526 (66.6%) and men 6288 (33.4%). Nurses saw 16238 (86.1%) and doctors 2612 (13.9%) of patients. The top 80 RFE and top 25 diagnoses are reported and ongoing care for hypertension was the commonest RFE and diagnosis. The 20 commonest RFE and diagnoses by age group are also reported.

**Conclusions/Significance:**

Ambulatory primary care is dominated by non-communicable chronic diseases. HIV/AIDS and TB are common, but not to the extent predicted by the burden of disease. Pneumonia and gastroenteritis are commonly seen especially in children. Women's health issues such as family planning and pregnancy related visits are also common. Injuries are not as common as expected from the burden of disease. Primary care providers did not recognise mental health problems. The results should guide the future training and assessment of primary care providers.

## Introduction

After the fall of Apartheid in 1994 the new government of South Africa committed itself to a district health system based on the principles of primary health care. Implementation of this policy involved the integration of fragmented health departments and the rapid expansion of access to care through building more clinics, making services free and placing the nurse practitioner on the front line. Nurses were supported by medical officers and more recently a new cadre of specialist family physicians has been introduced with responsibility for clinical governance, consulting more complex patients as well as mentoring and support of primary care providers.

The 2008 World Health Report *Primary Health Care – Now More Than Ever* reinforced the need for countries to implement primary health care. [1] In 2011 the South African government recognized that, while much has been achieved in terms of infrastructure and access to care, the country is still not getting value for money through its primary health care system. [2] For example South Africa is one of the few countries where infant mortality rates have been increasing despite the Millennium Development Goals and spending 8.3% of the GDP on health. [3] In light of this there is currently an active debate on the re-engineering of primary health care and an interest in the lessons that can be learnt from the Brazilian model and family health care teams. [4] If South Africa adopted such a model then primary health care teams would most likely consist of community health workers, professional nurses, clinical nurse practitioners and supporting medical officers or family physicians.

The South African burden of disease study has used disability adjusted life years (DALYs) to estimate the contribution of different diseases to mortality and morbidity at a community level. [5] The study supports the concept of a quadruple burden of disease with the largest burden derived from HIV/AIDS and TB. The other quadrants include interpersonal violence and trauma, maternal and child health issues and non-communicable chronic diseases. The burden of disease study has been invaluable in aligning health system planning and academic curricula with the needs of the country. However it does not tell us which of these conditions commonly present to primary care providers, how these conditions present or how providers make sense of them in terms of their diagnoses.

**Table 1:** Summary of sampling strategy.

Sub-district	Location	Population	Health workers	Required number of facilities				
				CHC		Clinic		Mobile
WESTERN CAPE PROVINCE								
Klipfontein	Urban	341,489	17	2	4		0	
Tygerberg	Urban	434,896	22	2	5		0	
Saldanha Bay	Rural	78,825	11	1	3		2	
Swartland	Rural	76,436	10	1	1		4	
LIMPOPO PROVINCE								
Ba-Phalaborwa	Rural	143, 410	12	1	3		1	
Greater Letaba	Urban	232,119	15	0	7		1	
Greater Tzaneen	Urban	393, 867	26	1	8		3	
Maruleng	Rural	98,565	7	0	3		1	
NORTHERN CAPE								
Sol Plaajtie	Urban	200,013	25	0	12		0	
Dikgatlong	Rural	39,881	17	0	9		0	
Magareng	Rural	21,348	6	0	3		0	
Phokwane	Rural	40,757	12	0	6		0	
NORTH WEST								
Ditsobotla	Rural	157,922	13	1	4		2	
Ramotshere Moiloa	Rural	142,417	14	1	4		1	
Ratlou	Rural	108,317	11	1	3		1	
Mafikeng	Urban	270,008	22	1	8		1	

*Community Health Centre.

As primary care is the first point of contact with the health services it could be expected that the conditions seen would be correlated with the burden of disease (as measured by DALYs). Where a different pattern is noted this maybe because these conditions do not present to the health services, present in vertical programmes or other levels of the health system and not primary care, or are poorly recognized when they do present.

Understanding the nature of presentations in primary care will greatly assist with the training of primary care providers and ensure that they are competent to assess the common undifferentiated symptoms. It will also enable the development of tools and educational resources.

Mismatches between the expected burden of disease and the actual presentations and diagnoses may also enable critical reflection on whether primary care is effectively engaging with the burden of disease and how the system should be modified for a better fit.

Previous studies that address these issues in South Africa have been on a small scale, focused on a single practice or health centre, or are outdated in terms of the current burden of disease. [6], [7], [8], [9]

This study aimed to determine the range and prevalence of reasons for encounter and diagnoses found among ambulatory patients attending public sector primary care facilities in South Africa.

## Methods

### Ethics statement

Ethical approval for the study was obtained from the Health Research Ethics Committees of the Universities of Stellenbosch and Cape Town and permission to conduct the study from the respective Provincial Departments of Health. Informed written consent was obtained from all health workers who participated in the study as approved by the ethics committees. Written consent was not required from the patients as no identifiers or additional information beyond that obtained in the usual consultation was collected and this was approved by the ethics committees.

### Study design

The study was a multi-centre prospective cross-sectional survey of consultations in primary care in four provinces of South Africa: Western Cape, Limpopo, Northern Cape and North West. These provinces were purposefully selected since research assistants, registered as postgraduate students for a Masters in family medicine, were available for fieldwork in the regions.

### Setting

In the South African setting about 16% of the population has insurance and makes use of the private sector. The remaining 84% of the population is dependent on the public sector, although some will pay cash for ad hoc use of the private sector.[10] The public sector primary care services are nurse-led with support from doctors. Primary care makes use of mobile clinics in remote areas to visit rural communities as well as fixed clinics. Clinics are usually only staffed by nurses and are themselves supported by larger community health centres. Community health centres are usually located in towns and urban areas and provide a wider range of health professionals and services, such as doctors, pharmacists, radiographers, or physiotherapists. Parts of the health centre may be dedicated to particular programmes or services such as HIV, TB or emergencies.

The provinces selected represent 32% of the South African population and range in size from the Northern Cape (1.8%) and North West (8.2%) to the Western Cape (10.2%) and Limpopo (11.7%). Professional nurses range from 89 per 100,000 population in North West to 119 per 100,000 in Limpopo; and for medical practitioners from 11 per 100,000 in North West to 32 per 100,000 in the Western Cape. The percentage of rural communities also ranges from 11% in the Western Cape to 89% in Limpopo.[11] These provinces, therefore, included large metropolitan areas as well as rural towns and remote farming communities. They included different climatic zones and some malarious areas.

### Sampling

The sample size was based on two considerations: firstly the number of health care workers a research assistant could train and support across a number of facilities and secondly on ensuring that the secondary reasons for encounter would be encountered in large enough numbers. The sample size per province was therefore the product of the number of health care workers that could be handled (60), the number of sampling days for each health care worker (5) and the number of patients per day (20) resulting in 6000 encounters per province and 24000 overall.

One district was purposefully selected from each Province based on the location of the research assistants. Out of these districts 4 sub-districts were purposively selected and at least 1 of the sub-districts was an urban area. Urban sub-districts were defined as having a town or metropolitan area and a population of more than 200,000 people. In the Western Cape sub-districts were selected from the Metropolitan and West Coast districts to enable a mix of rural and urban populations.

The sample size required from each sub-district to make up the total of 6000 for the Province was stratified according to the population of the sub-district. The facilities in each sub-district were then listed and divided into community health centres, fixed clinics or mobile clinics. It was assumed that a larger community health centre would have 5 health workers participating in the survey, a fixed clinic 2 health workers and a mobile clinic 1 health worker. It was also assumed that each health worker would see at least 20 patients a day and collect data on 5 separate days. The number of health workers required to deliver the sample size was then determined and distributed between the different types of facilities in proportion to the total number of different facilities in the sub-district (see Table 1). The required number of health centres, fixed clinics and mobile clinics were then randomly selected. In Tygerberg and Klipfontein the City of Cape Town, which runs the clinics, refused permission for the survey and therefore four community health centres were selected.

At each selected facility, health workers collected data on 5 separate days over a 1 year period. The first day was randomly chosen in the February-March period and then subsequent days booked every 2 months. Each of the 5 data collection days were also selected to be on a different day of the week so that each working day was covered once. This sampling strategy allowed for seasonal and daily variation in the patient presentations and diagnoses.

### Data collection

At each selected facility the research assistant explained the project and invited primary care providers, either doctors or nurses, to participate. Health workers received a small shopping voucher after each data collection day to thank them for their time and commitment. Health workers were provided with a data collection tool which allowed them to record the age and sex of each patient and up to 5 reasons for encounter and 5 diagnoses for that consultation. No distinction was made between primary and secondary or ongoing diagnoses. Data were collected on all sequential ambulatory patients seen by the health worker on that day. Health workers were expected to be working in general primary care and not a specialised vertical programme or emergency department.

### Data analysis

The International Classification of Primary Care Second Edition (ICPC-2) was used to code all reasons for encounter and diagnoses. [12] The ICPC-2 was developed by the World Organisation of National Colleges, Academies and Academic Associations of General Practitioners/Family Physicians (WONCA) as a classification system uniquely suited to primary care. The system enables classification of the reasons for encounter and diagnoses using a bi-axial structure. The first axis codes the body system involved by means of a letter derived from 17 possible chapters (Table 2). The second axis contains 7 components related to different aspects of the consultation as shown in Table 2. Within each component a menu of standardised rubrics are listed with definitions, inclusion and exclusion criteria. These rubrics provide a two-digit numeric code that is combined with the letter to give the final classification. For example HIV/AIDS is coded as B90, type 2 diabetes as T90, tuberculosis as A70.

**Table 2:** ICPC-2 bi-axial classification system.

Axis 1: Chapters based on body systems
A	General and unspecified
B	Blood, blood forming organs and immune mechanism (spleen, bone marrow)
D	Digestive
F	Eye
H	Ear (Hearing)
K	Circulatory
L	Musculoskeletal (locomotion)
N	Neurological
P	Psychological
R	Respiratory
S	Skin
T	Endocrine, metabolic and nutritional
U	Urological
W	Pregnancy, child bearing, family planning
X	Female genital
Y	Male genital
Z	Social problems

Axis 2: Components of the consultation
1	Complaints and symptoms
2	Diagnostic, screening and preventive activities
3	Medication, treatment and procedures
4	Test results
5	Administrative activities
6	Referrals and other reasons for encounter
7	Diagnosis/diseases (infectious, neoplasms, injuries, congenital, other)

The research assistants in each Province were trained to code the data using ICPC-2 and each provided an excel sheet with the consultations already captured and coded. The combined data were then analysed by the Centre for Statistical Consultation at Stellenbosch University. Descriptive statistics using frequency and means were calculated for the total data set, age groups, gender and provider type. The mean number of RFE and diagnoses for gender and provider type were compared by the Mann-Whitney U Test.

An error rate for each research assistant was analysed based on a representative random sample of their data sheets. The data sheets were coded independently by the principal researcher. The error rate for coding reasons for encounter was 11.3% (95%CI 7.4-15.2) and for diagnoses was 11.1% (95%CI 7.0-15.1).

## Results

Altogether 18856 consultations were included in the survey and generated 31451 reasons for encounter (RFE) and 24561 diagnoses. Limpopo provided 6678 (35.4%), Northern Cape 1504 (7.9%), North-West 5082 (26.9%) and Western Cape 5592 (29.6%) of the consultations. Women accounted for 12526 (66.6%) and men 6288 (33.4%) of consultations. Women presented with a mean of 1.65 RFE and men with significantly more at 1.69 (p<0.01). Primary care providers made a mean of 1.30 diagnoses in women and men.

Nurses saw 16238 (86.1%) and doctors 2612 (13.9%) of patients. Nurses had a mean of 1.65 reasons for encounter per consultation while doctors saw significantly more at a mean of 1.76 (p<0.05). Nurses made a mean of 1.24 diagnoses per encounter while doctors made significantly more at a mean of 1.69 (p<0.05).

**Figure 1:**
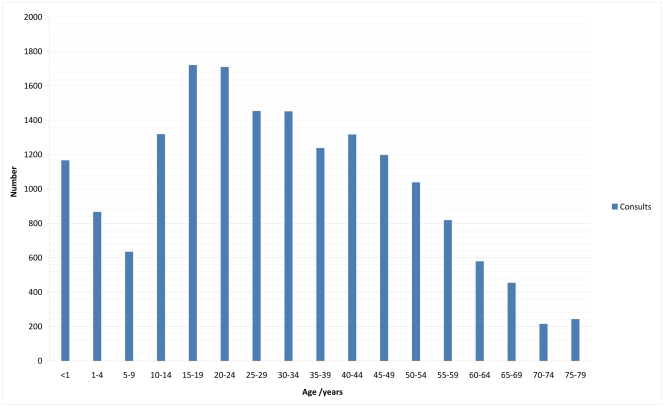
Distribution of number of consultations by age groups.

The distribution of the consultations with age is shown in Figure 1. The distribution shows two peaks, amongst infants and in the late teens/young adult age categories.

How the reasons for encounter were distributed between the different bodily systems in ICPC-2 are shown in Table 3. Patients in primary care mostly presented with respiratory, unspecified and cardiovascular problems. Although psychosocial problems are undoubtedly common amongst the population using the public sector, these were not commonly stated as the RFE. Neurological conditions were relatively common, but 1500 of these counts were due to headache alone.

**Table 3:** Distribution of RFEs between body systems in ICPC-2 (N = 31451).

ICPC-2 chapters	n	%
Respiratory	5499	17.5
General and unspecified	4521	14.4
Cardiovascular	3327	10.6
Digestive	2839	9.0
Musculoskeletal	2685	8.5
Pregnancy, child bearing, family planning	2489	7.9
Neurological	2303	7.3
Skin	1646	5.2
Endocrine, metabolic, nutritional	1565	5.0
Female genital	1108	3.5
Blood, blood forming organs and immune system	949	3.0
Urological	656	2.1
Eye	625	2.0
Ear	499	1.6
Psychological	398	1.3
Male genital	225	0.7
Social problem	117	0.4

Table 4 and Table 5 combined represent the 80 most common RFE and together also make up 26013 (82.7%) of all presentations in primary care. Nurse practitioners, as the first contact primary care providers, are expected to assess and manage these RFE. The top reason for encounter overall was ongoing care for hypertension.

**Table 4:** RFE associated with chronic care (N = 31451).

Reason for encounter	n	%
Cardiovascular e.g. hypertension (K31, K50, K61, K63, K64, K85)	2976	9.5
Women's health e.g. family planning, pregnancy (W14, W31, W50, W64)	2102	6.7
Immunisations (A44)	871	2.8
Unspecified e.g. TB (A50, A64)	892	2.8
Immune e.g. HIV (B34, B50, B60)	687	2.2
Metabolic e.g. diabetes (T50, T64)	618	2.0
Neurological e.g. epilepsy (N50)	248	0.8
Respiratory e.g. asthma (R50)	241	0.8
Psychological e.g. schizophrenia (P50)	183	0.6
Musculoskeletal e.g. arthritis (L50)	78	0.2

**Table 5:** Commonest complaints in primary care (N = 31451).

Reason for encounter	n	%
1. Cough (R05)	2821	9.0
2. Headache (N01)	1500	4.8
3. Fever (A03)	869	2.8
4. Sneezing/nasal complaint (R07, R08)	624	2.0
5. Sore throat (R21)	623	2.0
6. Back pain (L02, L03)	589	1.9
7. Generalised aches or pains (A01)	585	1.9
8. Diarrhoea (D11)	575	1.8
9. Abdominal pain or cramp (D01)	528	1.7
10. Dysuria (U01)	431	1.4
11. Loss of appetite (T03)	419	1.3
12. Vomiting (D10)	413	1.3
13. Leg or thigh pain or cramps (L14)	366	1.2
14. Generalised rash (S07)	318	1.0
15. Vaginal discharge (X14)	306	1.0
16. Vertigo/dizziness (N17)	299	0.9
17. Localised rash (S06)	290	0.9
18. Ear pain (H01)	281	0.9
19. Weakness/general tiredness (A04)	277	0.9
20. Pruritus (S02)	247	0.8
21. Abdominal pain, localised (D06)	232	0.7
22. Respiratory/pleuritic pain (R01)	223	0.7
23. Joint pain or symptoms (L20)	212	0.7
24. Knee pain or symptom (L15)	190	0.6
25. Shoulder pain or symptom (L08)	187	0.6
26. Shortness of breath (R02)	180	0.6
27. Chest pain (A11)	178	0.6
28. Foot and toe pain or symptoms (L17)	165	0.5
29. Weight loss (T08)	165	0.5
30. Swallowing problem (D21)	156	0.5
31. Hand and finger pain or symptom (L12)	154	0.5
32. Eye pain (F01)	151	0.5
33. Epigastric pain (D02)	134	0.4
34. Neck pain (L01)	132	0.4
35. Mouth, tongue, lip complaints (D20)	128	0.4
36. Eye discharge (F03)	126	0.4
37. Arm pain or symptom (L09)	121	0.4
38. Nausea (D09)	120	0.4
39. Menstruation absent/scanty (X05)	118	0.4
40. Sweating (A09)	116	0.4
41. Localized lump(s) or swelling(s) (S04)	116	0.4
42. Abnormal sputum (R25)	114	0.4
43. Respiratory complaint e.g. tight chest (R29)	110	0.3
44. Breathing problem (R04)	108	0.3
45. Genital/pelvic pain (X01)	107	0.3
46. Constipation (D12)	106	0.3
47. Ear discharge (H04)	100	0.3
48. Skin complaint (S29)	99	0.3
49. Red eye (F02)	96	0.3
50. Teeth or gum complaint (D19)	95	0.3
51. Chest pain, musculoskeletal (L04)	95	0.3
52. Trauma/injury (A80)	92	0.3
53. Vaginal symptoms (X15)	90	0.3
54. Eye sensation, abnormal (F13)	83	0.3
55. Heartburn (D03)	79	0.3
56. Urethral discharge (Y03)	78	0.2

Out of these 80 RFE Table 4 shows the distribution of follow up appointments for medication, examination and results. Chronic or ongoing care visits made up 8896 (28.4%) of these top 80 RFE. The second largest contributor to chronic care was women's health which included family planning and pregnancy related consultations. Chronic care for non-communicable chronic diseases made up at least 4344 (13.9%) of all reasons for encounter.

Out of the top 80 RFE Table 5 lists the commonest symptoms presented to primary care providers. Primary care providers need to have an approach to assessing and diagnosing patients who present with these undifferentiated complaints. Trauma and injury only compromised 92 (0.3%) of all RFE despite being the second largest contributor to the burden of disease. This would imply that trauma and injury is usually seen elsewhere, presumably in emergency rooms and hospital settings.

Table 6 shows the top 20 RFE by age group and allows a comparison between the under-5s, 5–14 years and 15 years and older.

**Table 6:** Top 20 RFE by age group.

	Under 5 years	N	%	5–14 years	N	%	15 years and older	N	%
	N = 2448	N	%	N = 3097	N	%	N = 2591	N	%
1	Cough R05	499	20.4	Women's health follow up W50, W64, W31	424	13.7	Cardiovascular follow up K50, K64, K31	2592	10.0
2	Fever A03	282	11.5	Cough R05	379	12.2	Cough R05	1943	7.5
3	Prevention/Immunisation A44	276	11.3	Headache N01	259	8.4	Women's health follow up W50, W64, W31	1354	5.2
4	General follow up e.g. TB A50 A64 A31	224	9.2	Sore throat R21	144	4.6	Headache N01	1231	4.8
5	Diarrhoea D11	122	5	Sneezing/nasal symptoms R07, R08	112	3.6	General follow up e.g. TB A50, A64	655	2.5
6	Loss of appetite T03	111	4.5	Vaginal discharge X14	30	3.6	Prevention/immunisation A44	591	2.3
7	Vomiting D10	85	3.5	Fever A03	106	3.4	General body pain A01	547	2.1
8	Sneezing/nasal complaint R07, R08	83	3.4	Abdominal pain general D01	85	2.7	Fever A03	481	1.9
9	Rash generalised S07	43	1.8	Diarrhoea D11	62	2.0	Sore throat R21	442	1.7
10	Rash localized S06	41	1.7	Rash generalised S07	55	1.8	Endocrine meds T50	421	1.6
11	Sore throat R21	37	1.5	Vomiting D10	53	1.7	Back symptom/complaint L02	413	1.6
12	Abdominal pain, general D01	32	1.3	Pruritus S02	45	1.5	Abdominal pain general D01	411	1.6
13	Breathing problem R04	31	1.3	Swallowing problem D21	44	1.4	Diarrhoea D11	391	1.5
14	Ear pain H01	30	1.2	Dysuria U01	41	1.3	Immunological follow up e.g. HIV B50	387	1.5
15	Mouth/tongue/lip complaint D20	23	0.9	Contraception W14	36	1.2	Dysuria U01	387	1.5
16	Eye discharge F03	21	0.9	General body pain A01	34	1.1	Leg/thigh symptom complaint L14	334	1.3
17	Shortness of breath R02	20	0.8	Ear pain H01	34	1.1	Contraception W14	279	1.1
18	Hair/scalp complaint S24	19	0.8	Rash localised S06	33	1.1	Loss of appetite T03	278	1.1
19	Ear discharge H04	17	0.7	Neurological follow up N50	30	1.0	Vomiting D10	275	1.1
20	Eye pain F01	16	0.7	Loss of appetite T03	30	1.0	Vertigo/dizziness N17	275	1.1

**Figure 2:**
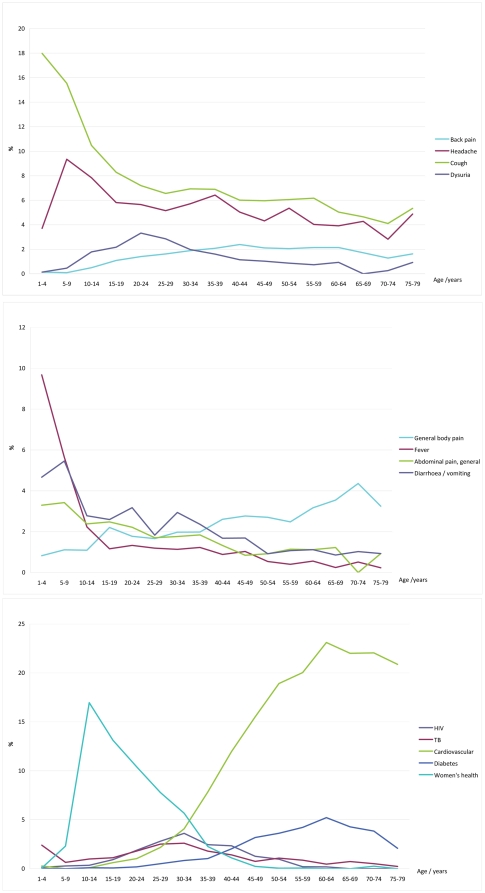
Patterns of selected reasons for encounter by age.

Figure 2 shows the pattern of selected RFE from the top 20 by age group. Figure 2a shows that cough peaks in the under-5s and then gradually declines, although it remains common in all age groups. Headache is a common symptom in all age groups. Dysuria is also found in all age groups with a small peak in the 20–24 year old bracket. Back pain gradually increases with age and becomes relatively stable as a symptom after the age of 30-years.

Figure 2b shows that fever starts at a peak in the under-5s, falls rapidly over the next 5-years and then levels out to decline more slowly over the adult years. Diarrhoea, vomiting and generalised abdominal pain or cramps follow a similar although less dramatic pattern. Generalised body pain is a constant feature in all age groups but tends to increase gradually with age.

Figure 2c shows that visits for women's health issues (family planning) peak in the 10–14 year old age group. HIV and TB peak in the 30–34 year old age group and TB also shows a peak in the under-5s. Cardiovascular (mostly hypertension) rises progressively from the teenage years to peak in the 60–64 year age group. Diabetes follows a similar pattern.

There were no major differences between the top 25 RFE between men and women apart from women's health visits for family planning, pregnancy and vaginal discharge.

The top 25 diagnoses are listed in Table 7 and represent 13065 (53.2%) of all diagnoses found in primary care. Hypertension is the commonest diagnosis by far, especially when uncomplicated and complicated cases are combined to give 3219 (13.1%) of all diagnoses. ICPC-2 does not provide codes for syndromic diagnosis of sexually transmitted infections and therefore the code for “Infectious disease, other” was used to code for sexually transmitted infection.

**Table 7:** Top 25 diagnoses in South African primary care (N = 24561).

Diagnosis	n	%
Hypertension, uncomplicated (K86)	2957	12.0
Upper respiratory tract infection (R74)	1306	5.3
HIV/AIDS (B90)	961	3.9
Type 2 diabetes (T90)	946	3.9
TB (A70)	862	3.6
Cough (R05)	681	2.8
Osteoarthritis (L91)	530	2.2
Gastroenteritis/diarrhoea (D73, D11)	491	2.0
Asthma (R96)	485	2.0
Acute tonsillitis (R76)	454	1.9
Epilepsy (N88)	375	1.5
Infectious disease, other (A78)	366	1.5
Urinary tract infection (U71)	317	1.3
Pneumonia (R81)	306	1.2
Acute bronchitis/bronchiolitis (R78)	263	1.1
Hypertension, complicated (K87)	262	1.1
Acute otitis media (H71)	233	0.9
Generalised body pain (A01)	213	0.9
Headache (N01)	209	0.9
Influenza (R80)	189	0.8
Muscle pain (L18)	183	0.7
Allergic reaction (A92)	176	0.7
Dermatophytosis (S74)	160	0.7
Chronic obstructive pulmonary disease (R95)	140	0.6

No psychiatric diagnoses appeared in the top 25 and the commonest diagnosis was schizophrenia (83, 0.3%). Depression (54, 0.2%) and anxiety disorders (19, 0.1%) were less commonly diagnosed than schizophrenia. Injury and trauma are also absent from the top 25 diagnoses. There were no significant differences in the top 25 diagnoses made in men and women apart from family planning and pregnancy amongst women and COPD amongst men.

Table 8 shows the top 20 diagnoses by age group.

**Table 8:** Top 20 diagnoses by age group.

	Under 5 year	N	%	5–14 years	N	%	15 years and older	N	%
	N = 1697	N	%	N = 2242	N	%	N = 20622	N	%
1	URTI R74	255	15.0	Contraception W14	303	13.5	Hypertension K86	2954	14.3
2	Health maintenance/prevention A98	169	10.0	URTI R74	202	9.0	Diabetes T90	942	4.6
3	Cough R05	121	7.1	Pregnancy W78	167	7.3	HIV B90	912	4.4
4	Pneumonia R81	113	6.7	Cough R05	125	5.6	Contraception W14	878	4.3
5	Immunisation A44	103	6.1	Acute tonsillitis R76	110	4.9	URTI R74	849	4.1
6	Diarrhoea D11	65	3.8	TB A70	57	2.5	TB A70	743	3.6
7	TB A70	63	3.7	Headache N01	46	2.1	Pregnancy W78	609	3.0
8	Acute otitis media H71	44	2.6	Epilepsy N88	41	1.8	Osteoarthritis L91	527	2.6
9	Acute tonsillitis R76	41	2.4	HIV B90	37	1.7	Asthma R96	446	2.2
10	Fever A03	40	2.4	Asthma R96	31	1.4	Cough R05	435	2.1
11	Gastroenteritis D73	35	2.1	Infectious disease, other A78	30	1.3	Health maintenance/prevention A98	354	1.7
12	Dermatophytosis S74	30	1.8	Pneumonia R81	30	1.3	Infectious disease, other A78	330	1.6
13	Impetigo S84	26	1.5	Influenza R80	28	1.2	Epilepsy N88	330	1.6
14	Vitamin/nutrition deficiency T91	25	1.5	Diarrhoea D11	27	1.2	Acute tonsillitis R76	303	1.5
15	Mouth/tongue/lip disease D83	23	1.4	Allergy/allergic reaction A92	26	1.2	UTI U71	289	1.4
16	No disease A97	22	1.3	Abdominal pain general D01	26	1.2	Hypertension, complicated K87	262	1.3
17	Allergy/allergic reaction A92	21	1.2	UTI U71	26	1.2	Acute bronchitis R78	225	1.1
18	Worms/other parasites D96	19	1.1	Gastroenteritis D73	25	1.1	Prevention/Immunisation A44	219	1.1
19	Influenza R80	19	1.1	Acute otitis media H71	23	1.0	General body pain A01	196	1.0
20	Conjunctivitis infectious F70	17	1.0	Acute bronchitis R78	22	1.0	Gastroenteritis D73	195	0.9

**Figure 3:**
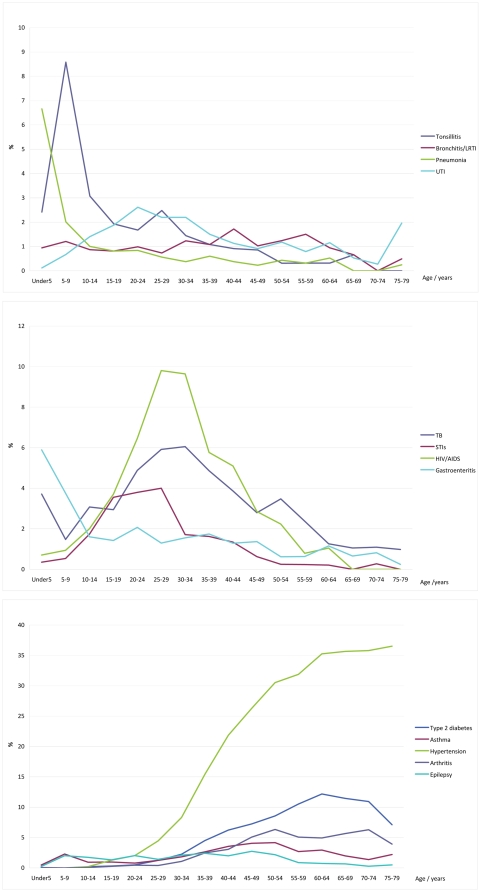
Patterns of selected diagnoses by age.

Figure 3a and 3b shows the distribution of common infectious diseases with age. Tonsillitis reaches a sharp peak in the 5–9 year old age group and then gradually declines. Gastroenteritis and pneumonia starts with a peak in the under-5 age group, then falls sharply over the next 5-years and afterwards slowly declines. Bronchitis and lower respiratory tract infections have a fairly constant frequency in all age groups. Urinary tract infections are present in all age groups but peak in the 20–24 year category. Sexually transmitted infections peak in the 25–29 year old age group and then drop sharply. HIV/AIDS and TB peak in the 25–34 year old age group.

Figure 3c shows the distribution of diagnoses for non-communicable diseases across age groups. Hypertension climbs continuously to reach a plateau in the 60–64 year age group. Asthma peaks in the 5–9 year old age group and then again in the 50–54 year old age group. Type 2 diabetes climbs to a peak in the 60–64 year old age group. Epilepsy has a constant presence from the 5–9 year old age group and reduces somewhat from 55–59 years onwards.

## Discussion

### Key findings

The findings reflect current morbidity found in South African ambulatory primary care. The late teens/young adult age groups predominate and this reflects the age distribution within the South African population. The adolescent population had amongst the highest number of consultations, which was a surprising result as this is usually a relatively healthy group. The high number of consultations appeared due to sexual health (contraception and pregnancy), HIV and related infections (STIs, TB, pneumonia, diarrhoea). Visits for contraception peaked in the 10–14 year age group. This finding may indicate that more attention be given to the special needs of adolescents in the design of health services as current programmes focus more on young children or adults.

Women accounted for a greater percentage of consultations than in other primary care settings [13] The reasons for this are unclear, but could be due to primary care taking more responsibility for reproductive health services or the higher prevalence of HIV amongst young women.[14]

The overwhelming majority of patients were seen by nurses. Doctors saw a much smaller percentage of patients, which is consistent with their usual role of just seeing patients referred to them by the nurses. Many clinics are only visited once a week by doctors. The higher mean number of RFE and diagnoses suggests that these patients had multi-morbidity and were probably more complicated than the patients seen by nurses.

If one compares the experience of primary care with the estimated burden of disease in South Africa there are some notable differences. HIV and TB as well as child health issues (pneumonia, diarrhoea) are well represented in primary care practice as they fall within the top 25 diagnoses. However as HIV is by far the leading cause of premature mortality and morbidity and TB the third leading cause one would have expected these to be more prominent in terms of diagnoses and chronic care visits.[5] The reason for this is probably because most patients with HIV and TB are not offered ongoing primary care, but are seen in specialised clinics and separate vertical programmes. Low birth weight and birth trauma/asphyxia are not seen, but would not be expected in an ambulatory primary care setting. Interpersonal violence (assault, injuries) and road traffic accidents which make a huge contribution to the burden of disease [5] are also rarely seen and will mostly likely present to emergency rooms and hospital settings. However the very low recognition of interpersonal violence as an issue is worrying as intimate partner violence is a large component of this in women and usually presents with psychological and other symptoms. [15] Non communicable chronic diseases are more prominent in primary care than expected from the burden of disease, especially hypertension and type 2 diabetes. Hypertension alone is the leading reason to attend primary care and the most common diagnosis, even in a context in which it is estimated that only 26% of men and 51% of women people are aware of their hypertension. [16] Mental disorders and substance abuse are not recognised or diagnosed, which is a major omission, as the WHO estimates that up to 24% of consultations in primary care include a mental disorder such as depression, anxiety or alcohol abuse. [17] The South African Stress and Health Survey estimated that 16.5% of people had a 12-month prevalence of a mental health disorder and that 26.2% of these were severe disorders. Depression, anxiety and alcohol use disorders were the commonest disorders found. [18] Problems such as deafness and cataracts, which appear in the top 20 contributors to the burden of disease, may also be poorly recognised.

The majority of patients were seen by nurses and not all were trained clinical nurse practitioners. Even clinical nurse practitioners only receive an additional 1-year of training to cope with the range of problems seen in primary care. The survey highlights the need to ensure that nurses are trained and competent to handle the common problems and raises the question of whether more consultations should be with doctors. The accuracy of their diagnoses cannot be determined from this data. However if nurses are expected to manage the range of diseases found in the survey they should also be enabled to treat them appropriately. For example in many provinces professional nurses are only allowed to prescribe hydrochlorothiazide for hypertension. The current revitalisation of primary care has to balance increasing availability of primary care services through nurse-led primary care teams with improving the acceptability and quality of those services. Family physicians and doctors may need to play a more active role in terms of mentoring and support.

A number of symptoms were used to provide diagnoses: cough, diarrhoea, generalised body pain, headache and muscle pain. In some cases this may represent an inability or unwillingness to make a more specific assessment or diagnosis. For example headache was rarely diagnosed in a more specific way such as tension headache or migraine. Generalised body pain is often a difficult presentation to make sense of or assess.

### Comparison to the literature

Compared to a similar survey performed in 2001–2 in the Eastern Cape of South Africa non-communicable chronic diseases and HIV/AIDS have both increased significantly amongst the reasons for attendance and diagnoses over the last 10-years. [7] For example cardiovascular reasons for attendance increased from fourteenth in 2001 to the third most common in 2010, while blood and immune reasons (mainly HIV) increased from seventeenth in 2001 to eleventh in 2010. It is surprising that HIV was not more prominent in both surveys. In 2001 the researchers believe this may have been due to a reluctance to record or diagnose it at that time while in 2010 it is most likely due to the treatment of HIV in separate vertical programmes.

When the reasons for encounter by ICPC chapter are compared with other countries such as the Netherlands, Poland, Japan and USA there are a number of similarities and differences. [13] Respiratory, digestive, skin, endocrine, female and male genital, urological, eye, ear and social reasons for encounter are similar in frequency. Psychological reasons for encounter are much higher in Netherlands and USA where they are within the top five chapters. Musculoskeletal complaints are also slightly higher in these other countries. Pregnancy and family planning related reasons for encounter are much higher in South Africa showing the important role that primary care plays in this area. Blood/immune (including HIV), general unspecified and neurological reasons are also higher in South Africa. Cardiovascular is similar across all countries except for the USA where it is much less a feature of primary care. There are many possible reasons for these differences including the health systems, cultural differences and the burden of disease.

When the top 52 symptoms/complaints from the Netherlands, Poland, Japan and USA are compared to the top 56 South African the majority are the same. [13] However in these other countries psychological complaints are found (feeling depressed, anxious, sleep disturbance) as well as complaints often associated with the elderly (vision problems, hearing complaints, blocked ears), which do not appear on the South African list. In contrast a number of complaints appear on the South African list that probably reflect the burden of disease from HIV/AIDS and TB (weight loss, sweating, loss of appetite, abnormal sputum, respiratory pain, dysphagia) and STIs (genital/pelvic pain, vaginal and urethral discharge, vaginal symptoms), which do not appear in these other countries. In addition infective complaints associated with the eye and ear (eye pain and discharge, red eyes, ear discharge), trauma/injury as well as absent or scanty menses are also listed. These may reflect the different burden of disease and more prominent role of primary care in pregnancy and family planning. The complaint of generalised/multiple body pain is also a particular feature of South African primary care and may reflect local cultural expressions of illness.

When the top 25 diagnoses are compared between these same countries and South Africa there is much less similarity. [13] In South Africa a number of diagnoses are found which do not appear in the top 25 from these other countries: HIV, TB, STIs, pneumonia, gastroenteritis, urinary tract infection, epilepsy and chronic obstructive airways disease. In these other countries the following diagnoses are found which do not feature on the South African list: sinusitis, osteoporosis, back pain, neck symptom/complaint, gastro-oesophageal reflux, peptic ulceration, gastritis, dermatitis, sleeping problem, depression, stroke, ischaemic heart disease, lipid metabolism disorder, laceration. This demonstrates quite substantial differences in the burden of disease encountered in South African primary care and the relatively high prevalence of infective and communicable diseases in South Africa compared to these countries. It again emphasises that mental health problems are under diagnosed. The prominence of COPD may be related to chronic lung disease from TB in addition to tobacco smoking.

### Strengths and limitations

The survey was not performed in all provinces of South Africa and it is possible that a different pattern could be found elsewhere. In addition districts and sub-districts were purposefully and not randomly selected within provinces, which could influence the results, should other sub-districts be significantly different, although this is not considered likely. Data from provinces was combined without stratification for differences in population size between provinces. This is the largest such survey performed in South Africa to date. Although the full sample size was not obtained the total number of consultations was sufficient to provide information on the prevalence of the commonest RFE and diagnoses. The sample size from the Northern Cape was a lot less than expected and was mainly due to a shortage of anticipated staff members to participate in the survey at each facility. The top RFE and diagnoses from the Northern Cape did not differ substantially from the rest of the survey and there is no reason to think that a larger sample would have changed the overall results. Errors in coding were often due to relatively minor differences, such as between R07 (sneezing/nasal congestion) and R08 (nasal symptoms/other) or to omissions such as when a recorded RFE was not coded. The article only reports on the commonest RFE and diagnoses where the error rate is likely to have less impact on the ranking of items. The accuracy of diagnoses cannot be determined.

### Implications and recommendations

The profile of primary care will inform the curriculum for training of primary care nurses, medical students and family physicians as this represents the presentations to which primary care providers must have an evidence based and effective approach. The profile should also influence the development of tools and content of educational resources, for example the recent expansion of the Practical Approach to Lung Health and HIV/AIDS guideline to include non-communicable diseases, mental health and antenatal care. The results highlight the need for more attention to psychological and social aspects of care in the training of primary care providers as well as the need for skills in ongoing and chronic care. The profile will also inform the assessment of these providers, for example in the exam offered by the College of Family Physicians.

The study provides useful feedback to district managers on the current focus of ambulatory primary care and can enable reflection on the direction of in-service training, allocation of resources and future organisation of care. It also reflects the vertical nature of HIV services, which exacerbates the problem of fragmented care for those surviving many years due to antiretroviral treatment, and who find themselves at increased risk of developing non-communicable disease and mental health problems. Already South Africa's Ministry of Health is exploring models whereby all chronic conditions, whether non-communicable, infectious or psychological could be integrated into a single chronic care service. Further analysis of the data set will be possible to explore what diagnoses primary care providers make from these presentations and to calculate the likelihood ratios of different conditions. For example how do primary care providers make sense of generalised body pain? It will also be possible to explore what presentations are commonly associated with specific diagnoses such as HIV/AIDS or depression and what diagnoses are commonly associated with each other.

### Conclusion

The survey presents a profile of morbidity in South African primary care and identifies the commonest reasons for encounter and diagnoses made. Ambulatory primary care is dominated by non-communicable chronic diseases such as hypertension and diabetes. HIV/AIDS and TB are present, but not to the extent predicted by the burden of disease, this is most likely because they are treated in separate vertical programmes. Pneumonia and gastroenteritis are commonly seen especially in children. Women's health issues such as family planning and pregnancy related visits are also common. Injuries are not as common as expected from the burden of disease and this is most likely because they present to emergency units. However it is also likely that intimate partner violence is unrecognised in primary care and providers appears to be failing to recognise and treat mental health problems such as depression and anxiety disorders. The results should guide the future training and assessment of primary care providers.
