# Correction: A Morbidity Survey of South African Primary Care

**DOI:** 10.1371/annotation/3545077e-aded-4eef-a460-be1edbd1845c

**Published:** 2012-05-15

**Authors:** Bob Mash, Lara Fairall, Olubunmi Adejayan, Omozuanvbo Ikpefan, Jyoti Kumari, Shaheed Mathee, Ronit Okun, Willy Yogolelo

There was an error in the affiliations of the third, fourth, fifth, sixth, seventh, and eighth authors. Olubunmi Adejayan, Omozuanvbo Ikpefan, Jyoti Kumari, Shaheed Mathee, Ronit Okun, and Willy Yogolelo are all affiliated with: 

1 Division of Family Medicine and Primary Care, Stellenbosch University, Cape Town, Western Cape, South Africa. 

